# Coinfection and repeat bacterial sexually transmitted infections (STI) – retrospective study on male attendees of public STI clinics in an Asia Pacific city

**DOI:** 10.1017/S0950268823000948

**Published:** 2023-06-09

**Authors:** Sze Long Chung, Ngai Sze Wong, King Man Ho, Shui Shan Lee

**Affiliations:** 1 Stanley Ho Centre for Emerging Infectious Diseases, The Chinese University of Hong Kong, Hong Kong, China; 2 JC School of Public Health and Primary Care, The Chinese University of Hong Kong, Hong Kong, China; 3 Department of Medicine, The University of Hong Kong, Hong Kong, China

**Keywords:** Sexually transmitted infection, Coinfection, repeat infection

## Abstract

Without protective immunity, recurrent sexually transmitted infections (STI) could occur. In this study, we retrospectively collected STI diagnosis records from public STI clinics attended by an average of 6,000 male patients annually in Hong Kong in 2009–2019. We estimated the prevalence of three bacterial STI (syphilis, chlamydia and gonorrhoea) coinfection from 2009 to 2019, and examined the factors associated with coinfection in 2014/15 and repeat infection in 2009–2019. We observed an increasing coinfection prevalence in male attendees with bacterial STI over the years, which reached the highest level of 15% in 2019. Among 3,698 male patients in 2014–2015, chlamydia/gonorrhoea coinfection was the commonest among all coinfections (77%). Factors such as young age (29 or below), HIV-positive status, and a history of concurrent genital warts/herpes were positively associated with coinfection in 2014/15 in multivariable logistic regression. Of all male patients with STI coinfection in 2014/15, those of age 30–49 and who self-reported as men who have sex with men (MSM) were more likely to have been repeatedly infected in 2009–2019. The results support the implementation of regular multi-STI testing as an STI control strategy for selected communities like MSM and people living with HIV.

## Introduction

Sexually transmitted infections (STI) constitute a major public health burden globally. The number of new cases of common curable STI is persistently high. The World Health Organization reported a total of 128 million cases of chlamydia, 82 million cases of gonorrhoea, and 7 million cases of syphilis in 2020 [1], of which males accounted for 46%, 56%, and 51% of the cases, respectively. Acquiring one STI may increase the chance of infection with another STI, for example, contracting syphilis or gonorrhoea was correlated with the likelihood of concurrent chlamydia [2]. Given that some STI carried mild or even no symptoms, asymptomatic concurrent infections could be missed if only symptom-based clinical testing was performed. Left untreated, undiagnosed patients might contribute to the increasing STI reservoirs [3], creating clusters and fuelling further epidemic spread. Evaluation of the characteristics of STI coinfection and repeat infection is important to facilitate early identification and timely treatment so as to reduce STI and HIV transmissions in the community.

Research on STI coinfection has been reported in many countries including India, Australia, Portugal, the Netherlands, and South Korea, which gave a highly varied prevalence ranging between 7% and 47% [4–8]. Most reported an equal or higher prevalence of coinfection in males than in females [4–7], showing that the phenomenon was particularly prevalent in males, who played a key role in sustaining transmission in the community. In Hong Kong, males accounted for the dominant STI burden [9], while the small number of female STI patients in public service were overrepresented by sex workers plus partners of male patients. Epidemiologic investigation of male STI coinfection would be important to enhance understanding of the underlying transmission dynamics.

Young age has been consistently shown to be associated with the risk of coinfection [4–7] as youngsters have a higher tendency to engage in risky sexual behaviours. An increasing trend of serosorting among men who have sex with men (MSM), that is choosing a sex partner with the same HIV status, was associated with an increased risk of acquiring bacterial STI due to reduced consistent condom use [10]. On a community level, sexually active MSM were often involved in diverse sexual networks [11] which may increase their chance of contracting multiple STIs repeatedly. On the other hand, heterosexual males with a spouse were found to be more likely to practise safer sex with casual or commercial partners than being single [12], as they might be more cautious about preventing transmission of STI to their spouse [13] after being infected once.

Most of the STI studies in the published literature were limited by their cross-sectional design without the collection of longitudinal data for assessment. Considering the sociodemographic variation across regions and the temporal changes in infection rates, the prevalence of coinfection and repeat infection and the underlying risk factors might vary. The current study was conducted to examine the pattern, prevalence, and factors associated with STI coinfection and repeat infection in males to enhance the epidemiological understanding of STIs in an Asia Pacific city, Hong Kong.

## Methods

### Patient sources and data processing

**Figure 1.**
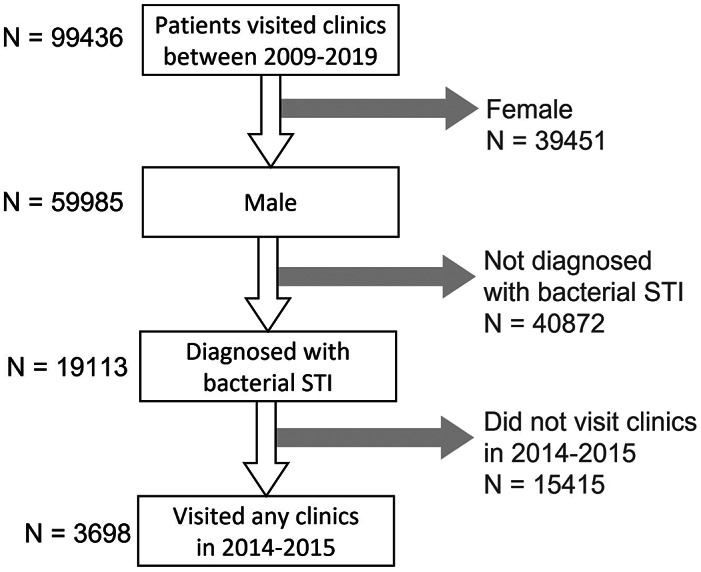
Selection process of the studied population.

**Figure 2.**
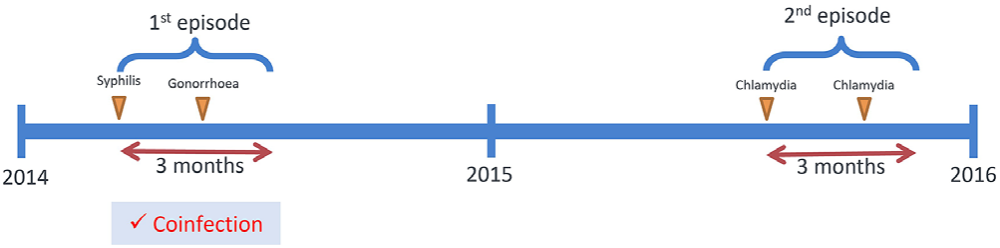
Illustration of the definition of coinfection.

In Hong Kong, Social Hygiene Clinics (SHC) are STI clinics in the public service that provide free STI examination, consultation, and treatment. The date of visit, diagnosis, socio-demographics, and sex partnerships of each attendee was recorded for each SHC attendance. Attendance records of male patients from all seven SHCs between 2009 and 2019 were retrieved retrospectively. Each patient had a unique patient ID which was transversal in all clinics. As an anonymous study, a study ID was assigned for each patient ID, and the mapping of study ID to patient ID was kept private by the clinic. The study ID was used to identify multiple visits made by the same patient. The number of male patients diagnosed clinically and/or microbiologically with bacterial STI, that is, syphilis, gonorrhoea, or chlamydia, in SHC each year was summarised. Data of male patients, who attended the SHC between 1 January 2014 and 31 December 2015, and had been diagnosed with any of these three bacterial STI, were selected for coinfection and repeat infection analysis (Figure 1) to ensure at least five years of follow-up. Coinfection was defined as when a case was diagnosed with more than one bacterial STI within three months, which was considered an infection within the same episode (Figure 2). The rate of coinfection referred to the proportion with coinfection among the patients diagnosed with any bacterial STI in the year. Repeat infection was identified when a patient had more than one episode of syphilis, chlamydia, or gonorrhoea diagnosis, not necessarily the same STI, between 2009 and 2019. Patients who self-identified as homosexual or bisexual were classified as MSM. Ethical approval was obtained from the Joint Chinese University of Hong Kong—New Territories East Cluster Clinical Research Ethics Committee (CREC) and data access was approved by the Department of Health, Hong Kong SAR government in compliance with the Personal Data (Privacy) Ordinance.

### Data analysis

In each year between 2009 and 2019, the number of male patients diagnosed with bacterial STI was summarised. Of these, the proportion who had coinfection within the year was calculated and plotted for graphical inspection. The confidence interval (CI) was calculated assuming Poisson distribution. A chi-square linear by linear association test was used to examine significant trends. Descriptive statistics were performed on included patients, to present the characteristics of male patients as stratified by coinfection status. All identified coinfection episodes in 2014–2015 were summarised according to the combination of STI.

Male patients’ age at the time of visit, ethnicity, marital status, sexual orientation, HIV status, commercial sex history, and genital warts and/or herpes diagnosis history were used as independent variables in bivariable logistic regression to analyse the factors associated with coinfections in each year. Subsequently, the patients who had visited the clinics between 2014–2015 were selected, stratified by coinfection status (coinfection VS single infection) to test if factors associated with repeat infection were different between the two groups. Factors significantly associated with the outcome variable in the bivariable model (p < 0.05) were entered into a multivariable logistic regression model (backward elimination). All analyses were performed in IBM SPSS. A complete case analysis was performed.

## Results

### Annual trends of SHC male attendees

**Figure 3.**
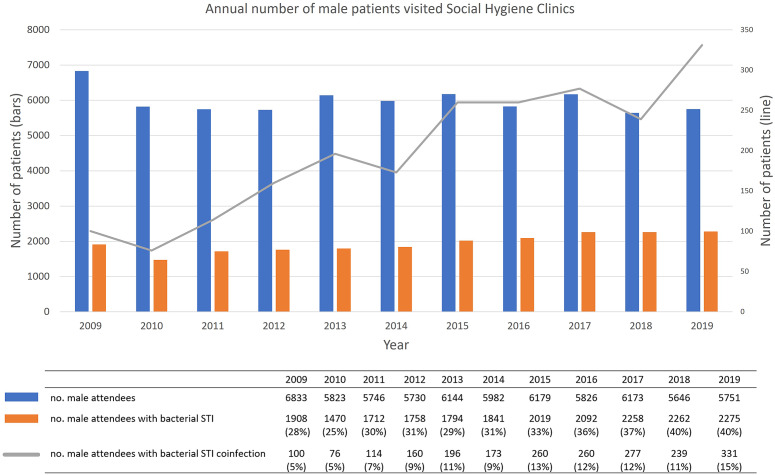
Annual number of male patients visited SHCs and number of male patients with coinfection in 2009–2019.

Between 2009 and 2019, 19,113 male patients that visited SHC have been diagnosed with bacterial STI. The annual number of male attendees remained approximately the same at around 6,000. During the same period, however, an increasing trend in the percentage of male patients with coinfection was observed (p < 0.01), reaching a high level of 15% (95%CI = 13%–16%) in 2019 (Figure 3). Each year, an average of 60% of patients were diagnosed with gonorrhoea while 33% were diagnosed with syphilis. An increase was observed in the number of male patients diagnosed with chlamydia (from 14% in 2009 to 33% in 2019) (Figure S1 in the Supplementary Material).

### General characteristics of male STI patients

A total of 3,698 male patients who had visited the clinics in the years 2014–2015 were diagnosed with syphilis, gonorrhoea, and/or chlamydia. Among them, the median age at the first visit in 2009–2019 was 32 (inter-quartile range (IQR) = 25–47), 90% were Chinese, 27% were married, 23% self-reported as MSM, 4% were HIV positive, and less than 1% reported ever having commercial sex within one year. Five hundred and eighty-six (16%, 95%CI = 15%–17%) of them had had coinfection, and 765 (21%, 95%CI = 19%–22%) had repeat infection.

Between 2014 and 2015, the most commonly diagnosed bacterial STI was gonorrhoea, which accounted for 52% of all diagnosed cases, followed by syphilis (37%) and chlamydia (25%). A total of 438 episodes of coinfection were recorded (Table 1), of which 14 episodes (3%) included all three infections. Chlamydia/gonorrhoea coinfection accounted for 77% of the coinfection episodes. Most of the coinfection episodes were detected in male patients aged 29 or below (53%), followed by 30–49 (38%) and 50 or above (10%).

**Table 1.** Distribution of STI combinations among coinfection episodes (N = 438)

Type of coinfection	N	%
2 infections		
Gonorrhoea + Chlamydia	338	77
Gonorrhoea + Syphilis	53	12
Chlamydia + Syphilis	34	8
3 infections		
Gonorrhoea + Chlamydia + Syphilis	14	3

### Factors associated with STI coinfection

In 2014–2015, the median age of coinfected male STI patients was 29.5 years old (IQR = 24–40), 72% of whom were single, 39% had a history of genital herpes or warts, 7% were HIV positive, and 30% self-reported as MSM. Five (1%) reported ever having commercial sex within the past one year. Married (single as reference, crude odds ratio (cOR) = 0.699, 95%CI = 0.555–0.881) patients had lower odds of coinfection after exclusion of MSM, while patients with repeat infection (cOR = 1.459, 95%CI = 1.190–1.790) were positively associated with coinfection in the univariable regression model. In the multivariable logistic regression model, male patients who were younger (aged 29 or below as reference, aged 30–49 adjusted odds ratio (aOR) = 0.760, 95%CI = 0.616–0.938; aged 50 or above aOR = 0.514, 95%CI = 0.390–0.677), or HIV positive (aOR = 1.910, 95%CI = 1.270–2.872), or had genital warts/herpes history (aOR = 9.044, 95%CI = 7.243–11.292) were more likely to have acquired coinfection than other male patients (Table 2).

**Table 2.** Characteristics and factors of the studied population associated with single infection (n = 3112) and coinfection (n = 586) in 2014–2015

Variables	Single infection, n (%)	Coinfection, n (%)	cOR (95%CI)	aOR (95%CI)
Age (median, IQR)	33, 26–49	29.5, 24–40		
29 or below	1194 (38)	293 (50)	reference	reference
30–49	1175 (38)	208 (36)	0.721* (0.594–0.877)	0.760* (0.616–0.938)
50 or above	743 (24)	85 (14)	0.466* (0.360–0.604)	0.514* (0.390–0.677)
Ethnicity				
Chinese	2801 (90)	517 (88)	reference	/
Non-Chinese	311 (10)	69 (12)	1.202 (0.911–1.586)	/
Marital status^#^				
Single	1553 (64)	294 (72)	reference	/
Married	869 (36)	115 (28)	0.699* (0.555–0.881)	/
HIV status				
Negative	3003 (97)	545 (93)	reference	reference
Positive	109 (3)	41 (7)	2.073* (1.431–3.002)	1.910* (1.270–2.872)
MSM (missing = 2)				
No	2442 (78)	409 (70)	reference	/
Yes	688 (22)	177 (30)	1.523* (1.253–1.853)	/
Ever had commercial sex within 1 year				
No	3093 (99)	581 (99)	reference	/
Yes	19 (1)	5 (1)	1.401 (0.521–3.767)	/
History of genital warts/herpes				
No	2914 (94)	360 (61)	reference	reference
Yes	198 (6)	226 (39)	9.239* (7.415–11.512)	9.044* (7.243–11.292)
Repeat infection				
No	2501 (80)	433 (74)	reference	/
Yes	611 (20)	153 (26)	1.459* (1.190–1.790)	/

*Note:* * p < 0.05; IQR = inter-quartile range; cOR = crude odds ratio; aOR = adjusted odds ratio; 95%CI = 95% confidence interval. # MSM was excluded in the univariable regression model for marital status.

Across the 10-year period, age remained a significant factor associated with coinfection (Table S1 in the Supplementary Material), with youngsters being more prone to coinfection. Being MSM and HIV positive was shown to be consistently positively associated with coinfection from the year 2014–2015 onwards.

### Factors associated with repeat infection

Among 3,698 male patients with bacterial STI diagnoses, 21% had repeat infection in 2009–2019. The median minimum interval between consecutive episodes of repeat infection was 14 months (421.5 days, IQR = 206–779 days), with 22% of cases occurring within 6 months, 22% within 7–12 months, 16% within 13–18 months, 11% within 19–24 months, 15% within 25–36 months, and 14% exceeding 36 months. Those with coinfection had shorter intervals between episodes (median = 293 days, IQR = 156.5–632 days) compared to those with single infection (median = 465 days, IQR = 224–805 days).

The median age of male patients with repeat infection was 31 years old (IQR = 26–41), out of which 9% were non-Chinese, 16% were married, 36% were MSM, and 6% were people living with HIV. Stratified by coinfection and single infection status in 2014/15, 26% (153/586) of patients with coinfection and 20% (611/3112) of patients with single infection reported repeat infection in 2009–2019. Among patients with coinfection in 2014/15, being aged between 30 and 49 (aOR = 1.568, 95%CI = 1.051–2.338) and self-reporting as MSM (aOR = 2.951, 95%CI = 1.969–4.422) were factors significantly associated with repeat infection in 2009–2019 (Table 3, Model A). In patients with single infection in 2014/15, being MSM (aOR = 1.613, 95%CI = 1.311–1.984), having a history of genital herpes/warts (aOR = 2.430, 95%CI = 1.788–3.302), and being married (aOR = 0.546, 95%CI = 0.429–0.694) were factors significantly associated with repeat infection in 2009–2019 (Table 3, Model B).

**Table 3.** Factors associated with repeat infection among patients with coinfection (n = 586) and single infection (n = 3112)

	Model A: Coinfection		Model B: Single infection	
Variables	cOR (95%CI)	aOR (95%CI)	cOR (95%CI)	aOR (95%CI)
Age (years old)				
29 or below	reference	reference	reference	/
30–49	1.568* (1.051–2.338)	1.686* (1.122–2.563)	0.991 (0.816–1.204)	/
50 or above	1.038 (0.587–.836)	1.508 (0.826–2.751)	0.479* (0.370–0.622)	/
Ethnicity				
Chinese	reference	/	reference	/
Non-Chinese	0.625 (0.332–1.178)	/	0.867 (0.639–1.178)	/
Marital status#				
Single	reference	/	reference	reference
Married	0.741 (0.420–1.305)	/	0.521* (0.409–0.665)	0.546* (0.429–0.694)
HIV status				
Negative	reference	/	reference	/
Positive	1.906 (0.988–3.675)	/	1.660* (1.085–2.542)	/
Self-reported MSM				
No	reference	reference	reference	reference
Yes	2.777* (1.887–4.085)	2.951* (1.969–4.422)	2.025* (1.665–2.463)	1.600* (1.300–1.969)
Ever had commercial sex within 1 year				
No	reference	/	reference	/
Yes	1.898 (0.314–11.471)	/	1.898 (0.718–5.014)	/
History of genital warts or herpes				
No	reference	/	reference	reference
Yes	1.203 (0.826–1.751)	/	2.770* (2.049–3.744)	2.430* (1.788–3.302)

*Note:* * p < 0.05; cOR = crude odds ratio; aOR = adjusted odds ratio; 95%CI = 95% confidence interval. # MSM was excluded in the univariable regression model but included in the multivariable regression model for marital status.

## Discussion

The analysis of 10 years’ data in this study shows that the rate of coinfection among male STI patients with bacterial STI in Hong Kong has tripled from 5% in 2009 to 15% in 2019. Worldwide, the percentage of STI coinfection among males has shown high variability between countries, with 47% in South Korea [8], 40% in India [4], 21% in Australia [5], 14% in Portugal [6], and 7% in the Netherlands [7]. The variation might have arisen from differences in the forms of STI investigated and definitions adopted for coinfection. For example, a study in South Korea included 12 pathogens which reported a 47% coinfection prevalence; while another study in the Netherlands summarised coinfection rate in terms of episodes/incidence which gave a lower coinfection rate of 7%. Nevertheless, our and others’ results suggested that coinfection is a phenomenon that is observed consistently in the population. In our study, chlamydia/gonorrhoea coinfection accounted for 77% of the episodes in Hong Kong, while other studies have also reported a high prevalence of gonorrhoea/chlamydia coinfection contributing to over 70% of all coinfection episodes [5, 7]. Other than the biological interactions between the two pathogens [14], the high prevalence may also be related to the detection methods as both pathogens were usually screened together by nucleic acid amplification tests (NAAT) in urine samples in reference to the Centers for Disease Control and Prevention (CDC) recommendations [15].

Younger age was positively associated with coinfection in male STI patients in our study, and this observation is consistent with other studies [4–7]. Those who were younger in age were usually sensation-seeking [16] and might have more sex partners and practise lesser condom use [17]. The widespread use of contraceptive measures other than condoms might have lowered condom use among some young people [18]. Similar to the results from a previous study, the higher proportion of coinfection in HIV-positive patients suggested persistent engagement in higher-risk sexual behaviours [19]. In our study, MSM was significantly associated with coinfection in the bivariable but not in the multivariable model. In our analyses, those who were younger in age (29 or below), were HIV positive, or had history of genital herpes or warts were also more likely to be MSM (results not shown). The nonsignificant association of MSM in the multivariable model could be due to the highly overlapping nature of these variables. Other than some behavioural characteristics, having a history of genital herpes or warts was also associated with coinfection. Patients might go for STI tests more frequently when genital herpes and warts recurred, which were symptomatic. While the diagnoses of genital herpes or warts could be associated with the transmission of other STIs [4,20], our results suggested that regular testing could have facilitated early identification of coinfections.

In Hong Kong, the combined prevalence of syphilis, gonorrhoea, and chlamydia was 2.7% in 2016 [21]. The prevalence of chlamydia among sexually active people aged 18–26 in 2016 was the highest at 5.3% compared to other age groups [21]. On top of that, those who were younger in age (25 or below) and who practised unprotected sex were at higher risk of chlamydia/gonorrhoea coinfection [22]. Higher percentage of repeat infection (26%) and the shorter interval between episodes among people with coinfection may imply that coinfection is a marker for persistent risky sexual practices [23]. Supplementing previous findings, the current study added to the epidemiologic profile of STI by the identification of factors associated with coinfection, suggesting that multi-STI testing would need to be considered as a strategy for selected communities like MSM and people living with HIV [24]. Regular STI testing in key populations even when asymptomatic may facilitate early identification and timely treatment for STI coinfection and thus reduce transmission. However, such intervention should be implemented with service support, as frequent testing may bring more asymptomatic patients to receive STI treatment. This may also lead to an increase in selective pressure, which may subsequently drive antimicrobial resistance in bacterial STI [25]. Studies suggest that a 6-monthly screening in a small, targeted population would be cost-effective in reducing STI prevalence over 10 years [26].

While the study had the advantage of multi-centre (7 clinics through the territory) data collection, there were a number of limitations. First, our data could only capture patients’ SHC records, while their visits to private clinics or hospitals were not collected. Indeed, the number of patients with repeat STI and HIV infection could have been underestimated if patients visited other clinics, such as HIV specialist clinics or private clinics, for managing repeat infections. Second, the adoption of the definition of coinfection with a 3-month window may have led to the overestimation of the number of coinfection episodes. The window was set based on the after-treatment test of the cure timeline recommended by the CDC [27]. There is still the possibility that a patient who had fully recovered from the first infection and contracted the second infection might have been classified incorrectly as having one episode within the 3-month window. Lastly, as a retrospective study with the inclusion of selected clinical data, STI treatment regimens and drug resistance records were unavailable in the same dataset, which made in-depth clinical correlative analyses not possible.

## Conclusion

All in all, our study has examined the situation of common STI coinfection in Hong Kong, and the results could provide a reference for epidemiology assessment in other cities in the Asia Pacific region. Although specific sexual behaviours were not explicitly studied, the characteristics of males at higher risk of coinfection and repeat infection have been identified, which may inform strategies for developing preventive intervention in the community. Coupled with the asymptomatic nature of some STI, our results supported the implementation of regular multi-STI testing and timely treatment for MSM and people living with HIV to not just facilitate early identification of STI but reduce ongoing transmissions in their network.

## Data Availability

The data that support the findings of this study are available from the authors upon reasonable request and following the procedure of government data access.

## Supplementary material

For supplementary material accompanying this paper visit http://doi.org/10.1017/S0950268823000948. click here to view supplementary material
