# Supplementary material for: Coinfection and repeat bacterial sexually transmitted infections (STI) – retrospective study on male attendees of public STI clinics in an Asia Pacific city
Source: Epidemiol Infect. 2023 Jun 9;151:e101. doi: 10.1017/S0950268823000948 (PMC10311681; doi:10.1017/S0950268823000948)
Supplement: Supplementary file 1 [file hygsup.zip › S0950268823000948sup001.docx]

**Supplementary Table 1. Bivariable logistic regression analysis of factors associated with coinfection stratified by year of visit.**

|  | Year | | | | | | | | | | | | | | | | | | | | | | |
| --- | --- | --- | --- | --- | --- | --- | --- | --- | --- | --- | --- | --- | --- | --- | --- | --- | --- | --- | --- | --- | --- | --- | --- |
|  | 2009 | | 2010 | | 2011 | | 2012 | | 2013 | | 2014 | | 2015 | | 2016 | | 2017 | | 2018 | | 2019 | |  |
|  | n | cOR | n | cOR | n | cOR | n | cOR | n | cOR | n | cOR | n | cOR | n | cOR | n | cOR | n | cOR | n | cOR |  |
| **Total no. of patients with bacterial STI** | 1908 |  | 1470 |  | 1712 |  | 1758 |  | 1794 |  | 1841 |  | 2019 |  | 2092 |  | 2258 |  | 2262 |  | 2275 |  |  |
| **Coinfection (%)** | 100 (5%) | | 76 (5%) | | 114 (7%) | | 160 (9%) | | 196 (11%) | | 173 (9%) | | 260 (13%) | | 260 (12%) | | 277 (12%) | | 239 (11%) | | 331 (15%) | |  |
| **Age Group** |  |  |  |  |  |  |  |  |  |  |  |  |  |  |  |  |  |  |  |  |  |  |  |
| 29 or below | 558 | 1 | 376 | 1 | 472 | 1 | 543 | 1 | 639 | 1 | 735 | 1 | 805 | 1 | 839 | 1 | 886 | 1 | 876 | 1 | 838 | 1 |  |
| 30-49 | 751 | **0.519 **** | 574 | **0.425 **** | 706 | **0.548  *** | 729 | **0.616**  ****** | 731 | **0.557**  ****** | 666 | **0.655 *** | 809 | 0.780 | 838 | **0.592 **** | 922 | **0.694 **** | 963 | 0.817 | 1024 | 0.901 |  |
| 50 or above | 598 | **0.311 **** | 520 | **0.329 **** | 534 | 0.805 | 486 | **0.468**  ****** | 424 | **0.435**  ****** | 440 | **0.214 **** | 405 | **0.393 **** | 415 | **0.186 **** | 450 | **0.236 **** | 423 | **0.41 **** | 413 | **0.174 **** |  |
| **Ethnicity** |  |  |  |  |  |  |  |  |  |  |  |  |  |  |  |  |  |  |  |  |  |  |  |
| Chinese | 1794 | 1 | 1377 | 1 | 1584 | 1 | 1597 | 1 | 1619 | 1 | 1652 | 1 | 1809 | 1 | 1896 | 1 | 2100 | 1 | 2053 | 1 | 2048 | 1 |  |
| Non-Chinese | 114 | 0.309 | 93 | 1.045 | 127 | 1.363 | 161 | 1.2 | 175 | 0.928 | 189 | 1.234 | 210 | 1.299 | 196 | 0.737 | 158 | 0.853 | 209 | 1.227 | 227 | 0.774 |  |
| **Marital Status#** | | | | | |  |  |  |  |  |  |  |  |  |  |  |  |  |  |  |  |  |  |
| Single | 1194 | 1 | 900 | 1 | 1082 | 1 | 1164 | 1 | 1256 | 1 | 1331 | 1 | 1514 | 1 | 1613 | 1 | 1691 | 1 | 1747 | 1 | 1786 | 1 |  |
| Married | 713 | **0.539 **** | 569 | **0.390 **** | 629 | 1.069 | 594 | **0.629 *** | 538 | **0.466 **** | 510 | **0.457 **** | 505 | **0.666 *** | 479 | **0.313 **** | 567 | **0.411 **** | 515 | 0.799 | 489 | **0.535 **** |  |
| **HIV Status** |  |  |  |  |  |  |  |  |  |  |  |  |  |  |  |  |  |  |  |  |  |  |  |
| Negative | 1879 | 1 | 1450 | 1 | 1676 | 1 | 1713 | 1 | 1723 | 1 | 1731 | 1 | 1877 | 1 | 1972 | 1 | 2141 | 1 | 2212 | 1 | 2211 | 1 |  |
| Positive | 29 | 0.642 | 20 | 0.965 | 36 | 1.282 | 45 | **3.845 **** | 71 | 1.192 | 110 | 1.442 | 142 | **1.918 **** | 120 | **1.736 *** | 117 | **3.504 **** | 50 | 1.389 | 64 | **1.836 *** |  |
| **Ever had Commercial Sex within 1 year** | | | | |  |  |  |  |  |  |  |  |  |  |  |  |  |  |  |  |  |  |  |
| No | 1907 | 1 | 1466 | 1 | 1711 |  | 1757 |  | 1794 |  | 1839 | 1 | 2007 | 1 | 2086 | 1 | 2257 | 1 | 2261 |  | 2271 | 1 |  |
| Yes | 1 | 0 | 2 | 0 | 0 | N/A | 0 | N/A | 0 | N/A | 1 | 0 | 12 | 0 | 6 | 1.411 | 1 | 0 | 0 | N/A | 3 | 0 |  |
| **History of Genital Herpes/Warts** | | |  |  |  |  |  |  |  |  |  |  |  |  |  |  |  |  |  |  |  |  |  |
| No | 1833 | 1 | 1412 | 1 | 1625 | 1 | 1625 | 1 | 1623 | 1 | 1652 | 1 | 1784 | 1 | 1857 | 1 | 2000 | 1 | 2151 | 1 | 2122 | 1 |  |
| Yes | 75 | 1.307 | 58 | **3.162 **** | 87 | 1.04 | 133 | 1.192 | 171 | 1.533 | 189 | 1.310 | 235 | **1.458 *** | 235 | **1.473 *** | 258 | 1.934 | 111 | **1.939 *** | 153 | 1.409 |  |
| **Self-reported MSM** | |  |  |  |  |  |  |  |  |  |  |  |  |  |  |  |  |  |  |  |  |  |  |
| No | 1819 |  | 1384 | 1 | 1592 | 1 | 1591 | 1 | 1537 | 1 | 1419 | 1 | 1506 | 1 | 1506 | 1 | 1601 | 1 | 1509 | 1 | 1418 | 1 |  |
| Yes | 87 | N/A | 79 | 0.709 | 118 | 1.486 | 167 | **2.114**  ****** | 257 | 1.043 | 420 | **1.424**  ***** | 513 | **1.606**  ****** | 586 | **1.603**  ****** | 657 | **2.091**  ****** | 753 | **1.631**  ****** | 857 | **2.106**  ****** |  |
| *Note*. *p < 0.05; **p < 0.01. # MSM was excluded in univariable regression model. cOR = crude odds ratio | | | | | | | | | | | | | | | | | | | | | | | |
