# Supplementary figures and images for: Coinfection and repeat bacterial sexually transmitted infections (STI) – retrospective study on male attendees of public STI clinics in an Asia Pacific city
Source: Epidemiol Infect. 2023 Jun 9;151:e101. doi: 10.1017/S0950268823000948 (PMC10311681; doi:10.1017/S0950268823000948)

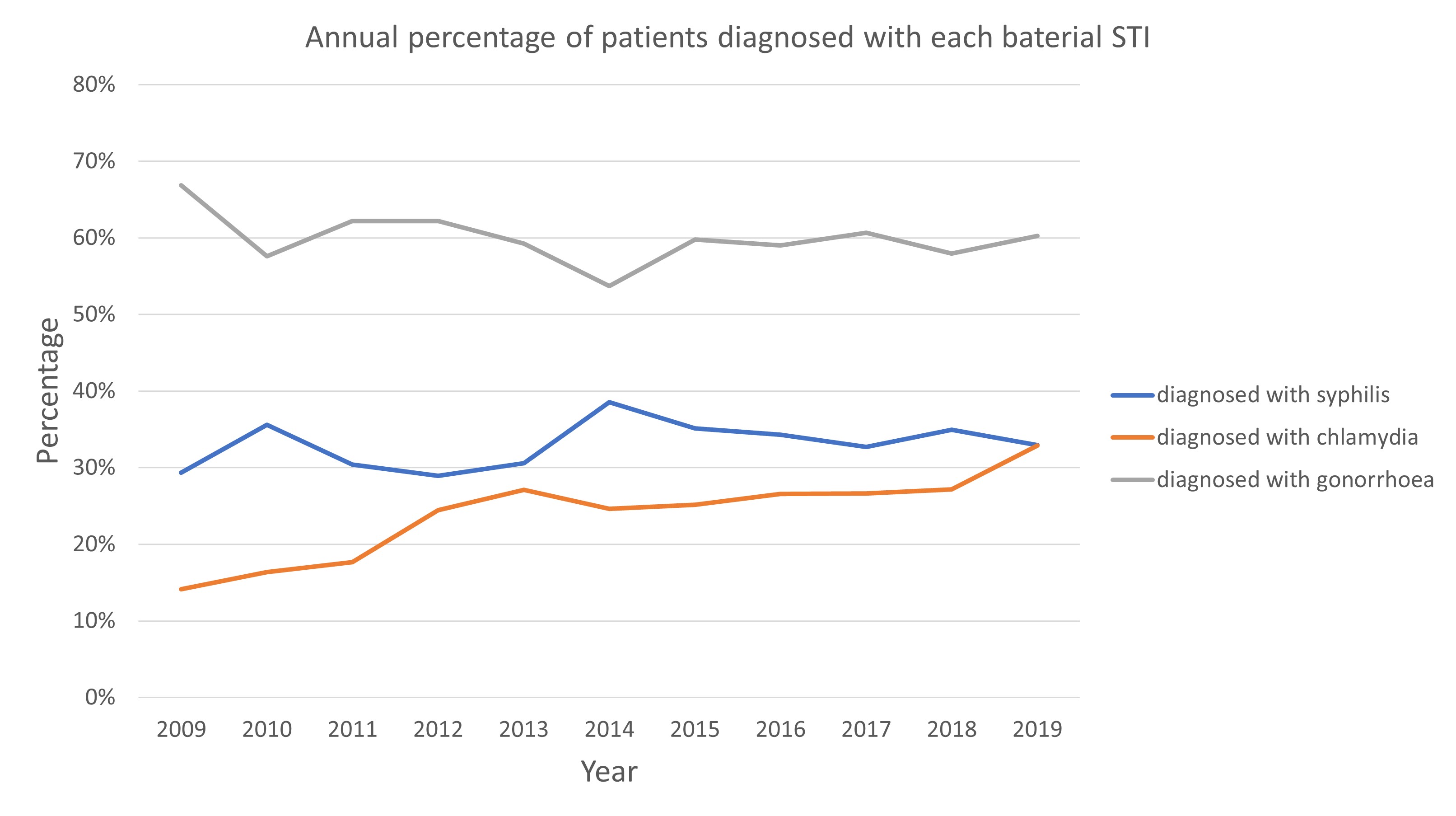

Supplement: Supplementary file 1 [file hygsup.zip › S0950268823000948sup002.jpg]
